# A call for national standardization and cut-off validation and not replacement of HbA1c

**DOI:** 10.1016/j.lansea.2026.100754

**Published:** 2026-03-21

**Authors:** Sutirtha Chakraborty

**Affiliations:** Department for Laboratory Medicine, Haugesund Hospital, Fonna Hospital Trust, Haugesund, Norway

We read with interest the article by Samajdar et al., which questions the reliability of HbA1c for diagnosing and monitoring diabetes in Indian populations.^1^ While the authors raise valid concerns regarding hematological confounders, we believe their conclusions overstate the case against HbA1c and insufficiently address the major limitations of the glucose based alternatives in diabetes diagnosis and management.

Firstly, the article overlooks a fundamental advantage of HbA1c (glycated haemoglobin) which has a superior biological variation (BV) profile over glucose. HbA1c exhibits a within subject BV and between subject BV of 1.2 and 5.4% respectively, compared with 4.7 and 8.0% for plasma glucose.^2^ This translates into substantially better test reproducibility. OGTT, which the authors propose as the gold standard, yields discordant results in 15–25% of individuals upon repeat testing a degree of irreproducibility that has serious consequences.^3^

Secondly, though the authors correctly point out the issues in HbA1c standardization issues in India where not all laboratories use methods traceable to NGSP or IFCC, there is no mention regarding standardization challenges with plasma glucose measurement. The glucose oxidase–peroxidase (GOD-POD) method, widely used in smaller laboratories from India due to its low cost, is susceptible to negative interference from ascorbic acid, uric acid, and bilirubin thereby producing falsely low glucose values. The hexokinase method, considered the IFCC reference, is costlier and less accessible and all cut-off points for diagnostic threshold are standardized with it. All glucose methods are not comparable with each other and the study by Dickson and colleagues has clearly shown the impact of this non agreement on estimation of prevalence of gestational diabetes.^4^

Thirdly, pre-analytical glycolysis reduces glucose concentration by approximately 5–7% per hour in unprocessed samples at room temperature and this creates large errors in settings where samples are transported from remote collection centres. HbA1c measured on whole blood is analytically stable and free from such problems and that too without the patient having to fast overnight or drink a concentrated glucose solution in case of OGTT (Oral Glucose Tolerance Test).^5^

While conditions such as iron deficiency anaemia, hemoglobinopathies, and G6PD (Glucose-6-Phosphate Dehydrogenase) deficiency are regionally prevalent, extrapolating their impact to the entirety of India's population is perhaps an overgeneralization.

Finally, while the multiparametric framework proposed in Table 3 includes haemoglobin electrophoresis, G6PD assays, iron panels, CGM (continuous glucose monitoring), and OGTT is academically well thought of, it may be impractical to implement in a lower-middle income country.

We therefore contend that HbA1c, measured using NGSP/IFCC certified methods like HPLC or Capillary electrophoresis which can simultaneously detect common haemoglobin variants and interpreted with nationally validated HbA1c cutoffs, remains the most scalable and reproduceable single diagnostic tool for diabetes diagnosis in India and other developing countries. The path forward for HbA1c is national standardization and clinical cut-off validation and not replacement.

## Contributors

Sutirtha Chakraborty: Conceptualization, Writing–original draft and Formal analysis.

## Declaration of interests

I declare no competing interests.
